# Thinking positively: The genetics of high intelligence

**DOI:** 10.1016/j.intell.2014.11.005

**Published:** 2015-01

**Authors:** Nicholas G. Shakeshaft, Maciej Trzaskowski, Andrew McMillan, Eva Krapohl, Michael A. Simpson, Avi Reichenberg, Martin Cederlöf, Henrik Larsson, Paul Lichtenstein, Robert Plomin

**Affiliations:** a King's College London, MRC Social, Genetic and Developmental Psychiatry Centre, Institute of Psychiatry, Psychology & Neuroscience, London, SE5 8AF, United Kingdom; b Department of Psychiatry, Mount Sinai School of Medicine, NY, 10029, USA; c Department of Medical Epidemiology and Biostatistics, Karolinska Institutet, Box 281, 17177 Stockholm, Sweden

**Keywords:** Intelligence, Human genetics, Twins, Siblings, Positive genetics

## Abstract

High intelligence (general cognitive ability) is fundamental to the human capital that drives societies in the information age. Understanding the origins of this intellectual capital is important for government policy, for neuroscience, and for genetics. For genetics, a key question is whether the genetic causes of high intelligence are qualitatively or quantitatively different from the normal distribution of intelligence. We report results from a sibling and twin study of high intelligence and its links with the normal distribution. We identified 360,000 sibling pairs and 9000 twin pairs from 3 million 18-year-old males with cognitive assessments administered as part of conscription to military service in Sweden between 1968 and 2010. We found that high intelligence is familial, heritable, and caused by the same genetic and environmental factors responsible for the normal distribution of intelligence. High intelligence is a good candidate for “positive genetics” — going beyond the negative effects of DNA sequence variation on disease and disorders to consider the positive end of the distribution of genetic effects.

## Highlights

• High intelligence is as familial and heritable as the rest of the distribution.
• There are no substantial genetic effects unique to high intelligence.
• There are no substantial environmental effects unique to high intelligence.
• Studying the “positive” end of trait dimensions is rare, and potentially very useful.

## 1 Introduction

High intelligence is precious human capital for advancing and maintaining society in the information age, as documented in studies that demonstrate that high intelligence is responsible for exceptional performance in many societally-valued outcomes (Kell, Lubinski, & Benbow, 2013; Lubinski, Benbow, Webb, & Bleske-Rechek, 2006; Rindermann & Thompson, 2011). Understanding the genetic and environmental origins of high intelligence is crucial for government policy (for example, for education in the STEM subjects of science, technology, engineering and mathematics), for neuroscience (for investigating the high-performance brain), and for genetics. A key question for genetic research is the extent to which the aetiology of high intelligence differs from the aetiology of the normal distribution of intelligence. More specifically, do the same genes affect both high intelligence and the rest of the distribution to the same extent? It cannot be assumed that the aetiology of high intelligence is the same. For example, very low intelligence (severe intellectual disability) differs aetiologically from the normal distribution, as proposed initially by Lionel Penrose (1938). In quantitative genetic studies (Nichols, 1984; Reichenberg et al., in preparation), a critical piece of evidence is that siblings of individuals with severe intellectual disability have an average IQ near 100, whereas siblings of those with mild intellectual disability have an average IQ of around 85, about one standard deviation below the population mean. In recent molecular genetic studies, rare non-inherited mutations appear to be a major source of severe intellectual disability (Ellison, Rosenfeld, & Shaffer, 2013).

One of the earliest studies in behavioural genetics was Galton's *Hereditary Genius* (1869), an analysis of family pedigrees for brains as well as beauty and brawn. Since there was no satisfactory way at the time to measure intelligence, Galton had to rely on reputation as an index of eminence, which he found to be highly familial. Since Spearman's (1904) seminal work on general cognitive ability (*g*) over a century ago, research has focused on intelligence as a general factor that indexes what diverse tests of cognitive abilities have in common (Jensen, 1998). Intelligence was the target of the first twin and adoption studies in the 1920s (Burks, 1928; Freeman, Holzinger, & Mitchell, 1928; Merriman, 1924; Theis, 1924), and continues to be among the most studied traits in behavioural genetics (Plomin, DeFries, Knopik, & Neiderhiser, 2013).

For these reasons, it is surprising that few behavioural genetic studies have focused on high intelligence (Plomin & Haworth, 2009). We review these studies below, but we begin with hypotheses about why genetic and environmental factors might differ for high intelligence (the Discontinuity Hypothesis), and why the results might be similar (the Continuity Hypothesis).

## 2 The **D**iscontinuity **H**ypothesis

The Discontinuity Hypothesis posits different environmental and genetic aetiologies for high intelligence in contrast to the rest of the distribution (Petrill, Kovas, Hart, Thompson, & Plomin, 2009). Although the evidence showing substantial heritability for the normal distribution of intelligence is one of the most consistently documented findings in the behavioural sciences (Deary, Johnson, & Houlihan, 2009), researchers in the field of expert training have argued that “differences in early experiences, preferences, opportunities, habits, training, and practice are the real determinants of excellence” (Howe, Davidson, & Sloboda, 1998, p. 403). A recent special issue of the journal Intelligence examines this environmental view of the acquisition of expertise (Detterman, 2014), including its relationship to genetic research (Plomin, Shakeshaft, McMillan, & Trzaskowski, 2014). Although the critical importance of deliberate practice is most often considered in the domain of specialist skills such as games, arts and sports, intelligence is also sometimes viewed as acquired expertise rather than inherited talent (Sternberg, 1999). If one accepts the overwhelming evidence showing substantial heritability for variation in the normal range of intelligence, the expert training position would suggest a discontinuity in the sense that it assumes that excellence is primarily due to environmental factors. Quantitative genetic research such as the twin method can test this hypothesis by investigating whether environmental influence is more important for high intelligence as compared to the rest of the distribution. Another more subtle environmental source of discontinuity can also be tested: the hypothesis that “differences in early experiences” are especially important for excellence would lead to the prediction that shared environment – environmental factors that make family members similar – should be greater for high intelligence.

Genetic reasons for discontinuity are also plausible, beginning with the folk wisdom that there could be “genes for genius.” The most persuasive case for genetic discontinuity for genius has been made by David Lykken (1998). He notes that a key problem of genius is “its mysterious irrepressibility and its ability to arise from the most unpromising of lineages and to flourish even in the meanest of circumstances” (p. 29). He proposed that genius emerges from unique combinations of genes; he referred to these higher-order nonadditive (epistatic) interactions as emergenic (Lykken, 1982, 2006). The emergenesis hypothesis does not necessarily predict that different genes affect high intelligence, but it does predict that genetic effects are nonadditive for high intelligence. The hallmark of an epistatic trait is one for which identical twins, who share all their genes, are more than twice as similar as fraternal twins and other first-degree relatives, who share on average 50% of their segregating genes. The twin design can test this hypothesis that nonadditive genetic effects are greater for high intelligence as well as testing the “genes for genius” hypothesis that different genes are responsible for high intelligence.

For both environmental and genetic discontinuity hypotheses, a crucial issue is the cut-off used to define high ability. If the cut-off is extremely high, scientific research gives way to case studies, as has been recently avowed by a leader in research on expert training, who advocated case studies of the “less than a handful of individuals… with the very highest levels of performance” (Ericsson, 2014). In genetics, too, there is interest in the very highest levels of performance. For example, Galton benchmarked the top 1 in a million (.0001%) as “illustrious” and the top 250 in a million (.025%) as “eminent” (Galton, 1869), and Lykken referred to “genius” although he did not suggest a specific cut-off. Such extreme cut-offs are beyond the reach of quantitative genetics research or gene-hunting research, both of which require large sample sizes. However, once genes accounting for at least a few percent of the variance at any level of performance are identified, they can be used with adequate power as a polygenic score in research on even “a handful of individuals with the very highest levels of performance” (Plomin & Deary, 2014). This is beginning to happen in the world of elite athletic performance where, contrary to the Discontinuity Hypothesis, the same genes appear to be associated additively with both ordinary and extraordinary performance (Epstein, 2013).

## 3 The **C**ontinuity **H**ypothesis

The Continuity Hypothesis posits that high performance is the quantitative extreme of the same environmental and genetic factors responsible for the rest of the normal distribution. From an environmental perspective, the prodigious practice and concentrated effort of high performers might be only quantitatively (e.g., number of hours of deliberate practice) but not qualitatively different from the factors responsible for the rest of the distribution. In terms of genetics, the Continuity Hypothesis is the foundation for quantitative genetic theory (Fisher, 1918). If multiple genes affect a trait, their joint effects are distributed as a normal bell-shaped curve, which means that the same genes affect the low and high extremes of such polygenic traits. Molecular genetic research has begun to confirm this polygenic prediction as genes are identified that contribute to the heritability of complex dimensions and disorders (Plomin, Haworth, & Davis, 2009). For example, genes identified by their association with obesity are associated with body weight throughout the distribution of weight (Speliotes et al., 2010).

## 4 Quantitative genetic analysis of high intelligence

When genes associated with intelligence are identified, they will provide a strong competitive test of these two hypotheses by assessing the extent to which genes associated with normal variation in intelligence are also associated with high intelligence and vice versa. Until that time, quantitative genetic methods such as the twin design can be used to compare the hypotheses. Quantitative genetic analyses have an advantage over molecular genetic approaches in terms of investigating environmental as well as genetic sources of continuity and discontinuity. For example, a twin study can test whether shared environmental influence is greater for high intelligence.

There are several ways that the twin method can be used to investigate whether genetic and environmental influences differ for high intelligence as compared to the rest of the distribution. These methods are described in greater detail in Methods section, but we introduce them here because of their relevance for reviewing previous studies of high intelligence. One set of methods uses a dichotomous “diagnosis” of high intelligence (case) or not (control). Monozygotic (MZ) and dizygotic (DZ) twin concordances can be compared to estimate genetic and environmental influence on high intelligence. Such dichotomous data are often analysed using a liability–threshold model, which assumes that liability is distributed normally until a threshold is exceeded, even though the analysis is based on dichotomous data (Rijsdijk & Sham, 2002). If the only available data were a “diagnosis” of high intelligence, the liability–threshold model is a useful way of assuming an underlying continuous liability despite having assessed a dichotomy.

Analysing high intelligence as a dichotomy loses much information when intelligence in the “cases” and “controls” has been assessed as a continuum. A method called DeFries–Fulker (DF) extremes analysis (DeFries & Fulker, 1985, 1988; DeFries, Fulker, & LaBuda, 1987) makes use of such quantitative trait data in estimating the genetic and environmental origins of the mean difference between the high intelligence group and the rest of the population. For this reason, heritability from DF extremes analysis is called group heritability to distinguish it from the usual estimate of heritability, which could be called individual differences heritability because it refers to genetic influence on individual differences throughout the distribution. Importantly, DF extremes analysis broaches the issue of the extent to which the same genes affect high intelligence and the rest of the distribution, as explained in Methods section.

## 5 Previous studies of high intelligence

Twin studies of high intelligence in childhood (Petrill et al., 1997; Plomin & Thompson, 1993; Ronald, Spinath, & Plomin, 2002) and in adulthood (Saudino, Plomin, Pedersen, & McClearn, 1994) have generally used DF extremes analysis and reported results consistent with the Continuity Hypothesis, in that group heritability was similar to individual differences heritability. However, the high-intelligence groups in these studies were small, just a few dozen pairs of twins, with the exception of one study (Ronald et al., 2002) which was limited by the age of the sample (2–4 years) and the measure (ratings of intelligence by parents). Low power to detect differences in heritability biases results in favour of the Continuity Hypothesis. Other studies have investigated the heritability of individual differences within high-intelligence groups, or asked more generally whether heritability differs across the population as a function of level of intelligence (Thompson, Detterman, & Plomin, 1993). However, such analyses address why one highly intelligent person is slightly more or less intelligent than another highly intelligent person, rather than asking why highly intelligent individuals as a group differ from the rest of the population.

In response to the neglect of research on high intelligence, the Genetics of High Cognitive Abilities (GHCA) Consortium was formed to bring together intelligence data on 11,000 twin pairs for the purpose of enabling an adequately powered comparison between high intelligence and the normal distribution. Liability–threshold model-fitting yielded evidence supporting the Continuity Hypothesis because estimates of genetic influence did not differ for high intelligence (0.50 with a 95% confidence interval of 0.41 to 0.60) and the entire sample (0.55; 0.51–0.59) (Haworth, Dale, & Plomin, 2009; Haworth et al., 2009). The overlapping confidence intervals suggest that heritability from the liability–threshold model in the high-intelligence group does not differ significantly from individual differences heritability. Estimates of shared environmental influence were also similar: 0.28 (0.19–0.37) for high intelligence and 0.21 (0.17–0.25) for the entire sample. However, the large confidence intervals for the high-intelligence group indicate that replication is needed to confirm the Continuity Hypothesis.

Finding similar heritabilities for high intelligence and the rest of the distribution does not confirm that the same genes are involved, which is the strength of DF extremes analysis. Moreover, in the GHCA study, only the top 15% were selected and the sample came from six twin studies each using different measures, in four countries, with a wide age range (6–71 years).

## 6 The present study

In contrast to the GHCA study, the present study used a higher cut-off (5%). It included non-twin siblings as well as twins. The sample was drawn from a single population and was assessed at the same age (18 years) on the same battery of cognitive measures, and the data were analysed with multiple methods including DF extremes analysis. Using a general factor from cognitive assessments of 3 million 18-year-old males administered as part of compulsory military service in Sweden between 1968 and 2010, we identified 370,000 sibling pairs and 9000 twin pairs. We selected the highest-scoring (top 5%) non-twin siblings and twins in order to investigate the familiality and heritability of high intelligence and its links to the normal distribution.

## 7 Methods

### 7.1 Sample

We tested the Continuity Hypothesis using cognitive assessments administered as part of military service in Sweden, from 1968 to 2010. Conscription was compulsory for males in Sweden until 2009, excluding those with severely disabling physical or psychiatric disorders, and achieved approximately 98% participation: 3 million 18-year-old males. From these, 363,905 families were identified containing at least two conscripted male siblings born in Sweden. From each family, we selected one twin pair if present (the youngest, if the family contained more than one pair); if there were no twins, we selected the two male siblings closest to one another in age (the youngest, again, if two such pairs had the same age difference). These selections were made using the Swedish Multi-Generation Register, which includes all individuals born in Sweden since 1932 or living in Sweden since 1961. The resulting data set comprised 3039 monozygotic (MZ) and 3196 dizygotic (DZ) twin pairs, 2780 twin pairs of unknown zygosity, and 354,890 pairs of non-twin brothers. The vast majority (96.7%) of the non-twin sibling pairs were separated in age by less than 2 years.

### 7.2 Measures

General cognitive ability was assessed with the Swedish Enlistment Battery (SEB), administered as part of the military conscription testing. Three different versions of the SEB were used during the 40-year period for which cognitive data were available: the SEB67 during the years 1970–1979, the SEB80 during 1980–1993, and the CAT-SEB during 1994–2009 (Carlstedt, 2000). The SEB67 and SEB80 were paper and pencil tests consisting of four subtests assessing verbal, visuospatial, technical and inductive abilities, which were summed to derive a measure for general cognitive ability. High internal consistency for the SEB80 has been reported (coefficient *α* = .79–.91) (Carlstedt & Mårdberg, 1993). Due to theoretical and methodological developments in intelligence research and the advent of the personal computer, a new version of the SEB (CAT-SEB), utilising computer-aided testing, was launched in 1994. The CAT-SEB was based on a three-level hierarchical model of cognitive abilities and included 12 tests, of which 10 were used to form the latent general ability factor, plus secondary factors of crystallised intelligence and general visualisation. The reliability of the CAT-SEB tests is also good (coefficient *α* = .70–.85) (Mårdberg & Carlstedt, 1998). The general cognitive ability variable, available from the Conscription Register and based on the different versions of the SEB, was measured on a stanine scale, i.e., a normally-distributed variable divided into nine levels (higher scores indicating greater ability), with a mean of 5 and standard deviation of 2.

### 7.3 Analyses

In addition to traditional individual differences analyses of the entire sample of twins and non-twin siblings (Plomin et al., 2013), two types of analysis were used for high intelligence: liability–threshold model-fitting using dichotomous data (high intelligence versus normal-range intelligence), and DeFries–Fulker (DF) extremes analysis, in which an “extreme” (or proband) group is selected (high-intelligence individuals, in this case), and quantitative variation in their siblings or co-twins is analysed. We begin with a brief description of other ways that have been used to analyse data of this type.

One general approach is to test for an interaction across the population between heritability (and environmental parameter estimates) and level of intelligence (Cherny, Cardon, Fulker, & DeFries, 1992; Logan et al., 2012). However, because there are relatively few individuals of high intelligence in the population, testing for an interaction throughout the entire population has little power to detect a difference in heritability specifically for high intelligence. Low power to detect interactions biases this approach in favour of the Continuity Hypothesis.

A more focused approach is to compare heritability for a high-intelligence group and an unselected group. A methodological problem with this apparently straightforward approach is that the variance of a high-intelligence group is restricted because they are highly selected, and this is likely to affect twin correlations. An important conceptual problem is that the focus of traditional heritability estimates is on individual differences. For understanding the origins of high intelligence, the issue is not whether one highly intelligent person is slightly more or less intelligent than another highly intelligent person, which is what is assessed in traditional heritability estimates. Instead, we are interested in the genetic and environmental causes of high intelligence — why highly intelligent individuals as a group differ from the rest of the population.

### 7.4 Liability–threshold model-fitting

The dichotomous data – high intelligence versus the rest of the distribution – can be analysed by comparing the degree of concordance for MZ and DZ twins, and for non-twin siblings. Here, we used probandwise concordance: the proportion of “affected” individuals (i.e., those with a stanine score of 9, in this case) who have a twin or sibling who is also affected. This method indicates morbidity risk, i.e., the probability that a sibling or co-twin of someone in the high-intelligence group will also be in that group.

Liability–threshold models assume that liability is normally distributed, but with the “disorder” (membership of the high-intelligence group, in this case) occurring only when a certain threshold is reached. Tetrachoric twin correlations and thresholds were calculated from our dichotomous data (Falconer, 1965; Smith, 1974), and liability–threshold (and all other) model-fitting analyses were conducted using OpenMx (Boker et al., 2011). This model-fitting produces ACE estimates analogous to those produced by twin model-fitting for continuous data, but the heritability estimate is the heritability of a hypothetical continuous liability construct, derived from the dichotomous data.

### 7.5 DeFries–Fulker (DF) extremes analysis

Analysing continuous data as dichotomous loses a great deal of information. Here, intelligence is assessed as a continuous stanine (standardised, nine-point) score, so much more information is available than the dichotomised “diagnosis” of high intelligence assessed by liability–threshold modelling.

We can use these continuous data to estimate the genetic and environmental origins of the mean difference between the high-intelligence group and the rest of the distribution, using DF extremes analysis (DeFries & Fulker, 1985, 1988; DeFries et al., 1987). This technique assesses the degree to which the co-twins or siblings of the extreme (high intelligence) group regress to the population mean. If the co-twin/sibling mean differs from the population mean, the trait is familial. Further, if the mean for MZ twins regresses less than that for DZ twins (and non-twin siblings), this indicates genetic influence on the mean difference between the high-intelligence group and the rest of the population.

In DF extremes analysis, the trait scores are standardised and transformed to account for the mean differences between the MZ and DZ groups, then fitted to the regression equation: *C = β*_1_*P + β*_2_*R + A*. *C* is the predicted score for the co-twin; *P*, the proband score; *R*, the coefficient of genetic relatedness (1.0 for MZ twins, 0.5 for DZ twins and non-twin siblings) and *A*, the regression constant. *β*_1_ is the partial regression of the co-twin score on the proband score, and represents the average twin resemblance, independent of *β*_2_. *β*_2_ is the partial regression of the co-twin score on *R* independent of *β*_1_, and is equal to double the difference between the MZ and DZ co-twin means (adjusted for any differences between MZ and DZ probands). Dividing *β*_2_ by the difference between the proband and population means provides the “group heritability,” the proportion of the difference between the proband and population phenotypic means that is genetic in origin. (This should be contrasted against the usual heritability estimates produced by traditional twin model fitting analyses, which represent the genetic influence on individual differences, rather than the influence on the mean difference between probands and the rest of the population.)

A finding of group heritability indicates that both the extreme trait and the rest of the distribution are heritable. Importantly, however, it also indicates that the genetic contributions in both cases are not independent from one another: the group heritability for two heritable but unrelated traits would be zero (Plomin & Kovas, 2005). In effect, DF extremes is a bivariate analysis: in this case, between the extreme score and the rest of the quantitative dimension. Finding substantial group heritability thus indicates not only that both the dimensional trait and its quantitative extreme are heritable, but also that they are influenced in part by the same genes: extreme scores are not qualitatively distinct from the rest of the distribution.

## 8 Results

This study assessed the genetic architecture of high intelligence. We present the results of classical twin model-fitting for the whole distribution of intelligence. For high intelligence vs. the rest of the distribution, we present twin and sibling pair concordances, liability–threshold model-fitting analyses of dichotomous twin data, and DF extremes analyses incorporating quantitative twin data. First, we provide descriptive results and a simple representation of the familiality of high intelligence.

### 8.1 Descriptive statistics

**Fig. 1:**
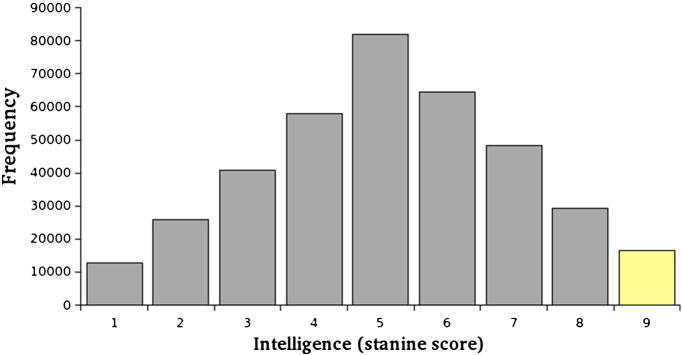
Distribution of intelligence scores. *N* = 363,905, mean = 5.16, SD = 1.94. Data shown include one randomly-selected individual per sibling pair. The highest-scoring individuals (stanine 9) are highlighted (*N* = 16,058).

Fig. 1 shows the distribution of stanine scores for intelligence for the total sample described above, selecting one sibling at random from each pair. As indicated, the data are normally distributed (mean = 5.16, SD = 1.94), with 5% of individuals achieving the highest possible stanine score (9), corresponding to an IQ above 125. The siblings of these probands were selected, comprising a sample of 185 MZ twins, 196 DZ twins, and 28,339 non-twin siblings.

The aim of this study is to estimate the genetic and environmental influences accounting for the difference (amounting to 1.98 standard deviations) between the highest scoring individuals and the population mean. As explained above, we are not concerned with the individual differences between the highest scoring individuals themselves (which cannot be assessed in any case, as only stanine scores are available for this sample), but rather with the differences between this group as a whole and the rest of the population.

For subsequent analyses, to account for any changes in population mean intelligence over time, the raw stanine scores were regressed on year of birth, and standardised.

### 8.2 Individual differences (whole twin sample)

Before exploring the differences between the high-intelligence group and the rest of the population, the twin sample as a whole was analysed to confirm the validity and representativeness of these data and to provide a comparison for the analysis of high intelligence. The correlation between scores for MZ twins was 0.80, and for DZ twins 0.51, which suggests heritability of 0.58 for intelligence in this sample (by doubling the difference between these correlations to produce a rough estimate) and is inconsistent with nonadditive genetic effects (as the MZ correlation is less than double the DZ correlation). More rigorous estimates, produced by univariate ACE twin model-fitting, are presented in Table 1. This analysis partitions variance in the sample's scores into additive genetic (A), shared environmental (C) and non-shared environmental (E) components.

These results, suggesting substantial genetic influence, with environmental influences evenly divided between shared and non-shared effects, correspond very closely to those typically found in the literature for participants of this age (Haworth et al., 2010). This suggests that these data are in line with those obtained by other studies.

### 8.3 Familiality of high intelligence

The familiality of high intelligence can be observed simply by comparing the mean scores of the non-twin siblings of high-intelligence probands to the population mean.

**Fig. 2:**
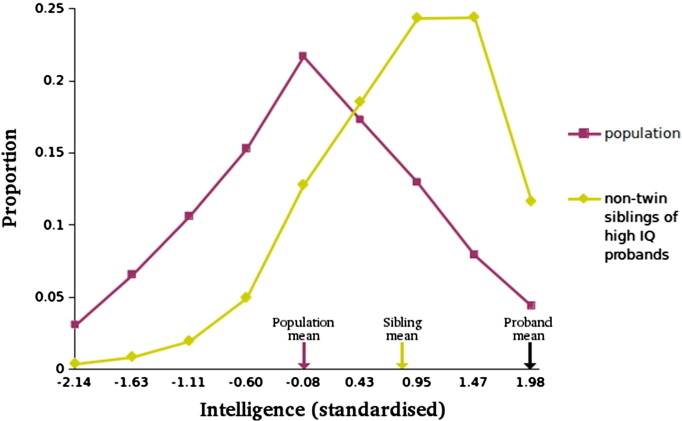
Familiality of high intelligence. Male siblings of high-intelligence probands (with a standardised score of 1.98) have significantly and substantially higher intelligence (mean = 0.81, SD = 0.81, *N* = 28,339) than the population (mean = 0, SD = 1, *N* = 727,810).

As shown in Fig. 2, high intelligence, defined as the highest 5% of scores, is highly familial. For siblings of probands (i.e., those with a standardised score of 1.98, equivalent to a raw stanine score of 9), the distribution of intelligence is shifted sharply to the right of that of the rest of the population, with a mean score (0.81) approximately halfway between the proband score and the population mean (0). These results suggest a sibling “group correlation” (the ratio between the siblings' deviation from the population mean to the probands' deviation from the population mean) of 0.41. In other words, almost half of the difference between high intelligence and the rest of the population is familial in origin.

**Fig. 3:**
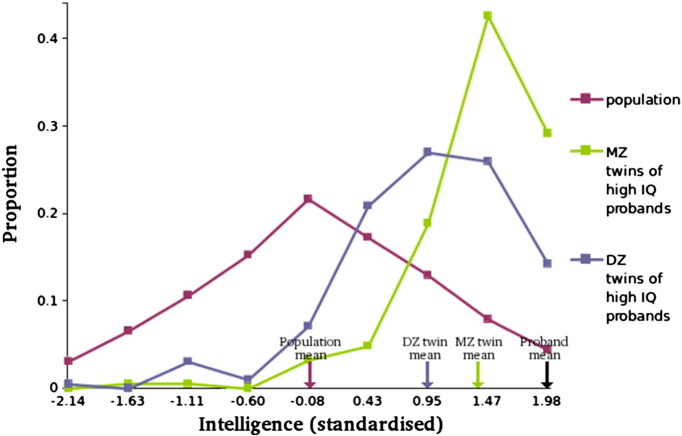
Heritability of high intelligence. Male MZ co-twins of high-intelligence probands (with a standardised score of 1.98) have significantly and substantially higher intelligence (mean = 1.39, SD = 0.58, *N* = 185) than DZ co-twins (mean = 0.95, SD = 0.75, *N* = 196), who in turn score significantly and substantially higher than the population (mean = 0, SD = 1, *N* = 727,810).

Familiality could be due to genetic or environmental influences. However, the twin data presented in Fig. 3 indicates that the familial effect is substantially genetic in origin. The mean for DZ twins of probands (0.95) does not differ substantially from that of non-twin siblings, as shown in Fig. 1. In contrast, MZ co-twins have a substantially higher mean score (1.39) than that of DZs, suggesting a strong genetic association.

More rigorous and specific results can be obtained, as described below.

### 8.4 Dichotomous data: Concordances

Table 2 presents twin/sibling concordances for the high-intelligence MZ twin, DZ twin and non-twin sibling groups.

For the 28,339 selected non-twin sibling pairs, there were 6604 individuals in 3302 concordant pairs, and 50,074 individuals in 25,037 discordant pairs. Simple (pairwise) concordance is thus 12% (i.e., 3302/28339, the proportion of pairs that are concordant). However, probandwise concordance is a better measure, since it indicates morbidity risk. For these siblings, probandwise concordance is 21% (i.e., (2*3302)/((2*3302) + 25037)), which is the probability that the twin or sibling of a proband will also be a proband. These results indicate substantial familiality for high intelligence.

For twins, the same calculations indicate probandwise concordance of 45% for MZ twins, and 25% for DZ twins. In other words, there is a 45% probability that the MZ twin of an individual in the high intelligence group will also be in that group (and 25% for a DZ twin). Doubling the difference between the MZ and DZ concordances would suggest heritability of 0.40 — but as these concordances do not take account of population base rates, this is not entirely appropriate statistically. Tetrachoric and group correlations (presented below) are preferable for this reason.

All subsequent analyses (tetrachoric correlations, liability–threshold model-fitting and DF extremes analysis) were conducted using the full twin sample of 6235 pairs.

### 8.5 Dichotomous data: Liability–threshold model-fitting

As discussed in Methods, dichotomous data liability–threshold modelling may be used to analyse dichotomous data, assuming that liability (i.e., the “risk” of high intelligence, in this case) is normally distributed, but a certain threshold must be exceeded for an individual to become a proband. The liability–threshold model is based on twin tetrachoric correlations, which are presented in Table 3.

These tetrachoric correlations, derived from dichotomous data, may be analysed in the same way as twin correlations from continuous data. For example, doubling the difference between the MZ and DZ correlations suggests heritability of 0.44 for high intelligence. As with the twin correlations for the whole distribution, these results do not suggest the existence of nonadditive genetic effects. Liability–threshold model-fitting provides a more rigorous analysis. Results are presented in Table 4.

These results suggest substantial heritability (0.42), with environmental influences divided between shared and non-shared effects. All of these variance components were significant, and an analysis of sub-models (eliminating variance components and testing the decrease in fit to the data) indicated that this full model best fit the data. As noted in Methods, however, these results refer to the variance of a hypothetical construct of continuous liability for high intelligence, derived from the dichotomous data, rather than that of a quantitative, continuous measure of intelligence.

### 8.6 Continuous data: DeFries–Fulker (DF) extremes analysis

As shown in Fig. 3, MZ co-twins of those in the high intelligence group regress to the population mean to a much smaller extent than do DZ co-twins, suggesting genetic influence. As discussed in Methods, DF extremes analysis uses continuous data, and can estimate the genetic and environmental factors influencing the difference in mean intelligence between the two intelligence groups (high intelligence vs. the rest of the population), by quantifying the differential regression to the mean for MZ and DZ co-twins of probands.

The process can be illustrated without using model-fitting, as shown above for non-twin siblings (“Familiality of High Intelligence”). Whereas a conventional twin correlation refers to individual differences on a trait, a “group” correlation quantifies the mean difference between the extreme group (i.e., the high intelligence group, here) and the rest of the population (Plomin, 1991). This may be calculated as the ratio between the two groups' differences from the mean, i.e., that of the probands and that of their co-twins. (In animal selection studies, these are known as the “selection differential” and “response to selection,” respectively; Plomin et al., 2014.) For these data, this yields group correlations of 0.70 for MZ twins, and 0.48 for DZ twins. Doubling the difference between these group correlations estimates group heritability at 0.44, suggesting that almost half of the mean difference between the high intelligence group and the rest of the population is explained genetically.

DF extremes model-fitting is preferable, because it uses the full twin data set (6235 pairs), and does not rely on randomly selecting one member of each concordant pair. It would also take into account any mean differences between MZ and DZ probands, although there are none with these stanine data. The DF extremes model-fitting results are presented in Table 5.

The DF extremes group heritability estimate (0.40) is similar to that estimated using the simpler group method above, and to the liability–threshold model-fitting results. The close approximation between the DF extremes and liability–threshold model-fitting results suggests that the assumptions of the latter are correct (Plomin & Kovas, 2005).

As with the previous analyses using dichotomous data, the DF extremes results indicate that just under half of the mean difference between the high intelligence group and the rest of the population is explained genetically, with the remaining variance divided between shared and non-shared environmental influences.

## 9 Discussion

These results provide strong support for the Continuity Hypothesis. Familial resemblance from non-twin sibling analyses and heritabilities from twin analyses were similar for high intelligence and for the rest of the distribution, using concordances, liability–threshold analysis, and DF extremes analysis. As explained earlier, DF extremes analysis not only indicates substantial heritability of high intelligence and of individual differences in intelligence in the normal distribution but also suggests substantial genetic correlation between them. Importantly, our twin results are highly similar to the results of the only other large twin study of high intelligence (GHCA; Haworth, Wright, et al., 2009).

For these reasons, we conclude that high intelligence is familial, heritable, and caused by the same genetic factors responsible for the normal distribution of intelligence. Stated more provocatively, high intelligence as we defined it appears to be nothing more than the quantitative extreme of the same genetic factors responsible for normal variation.

We found no support for the genetic Discontinuity Hypothesis that nonadditive genetic variance is greater for high intelligence, as suggested by the emergenesis hypothesis (Lykken, 1982, 2006). There was no evidence for nonadditive genetic variance for either high intelligence or for the entire sample, which is similar to GHCA results. One caveat concerns assortative mating. Assortative mating is much greater for intelligence (spouse correlations ~ 0.40) than for personality (spouse correlations ~ 0.10) or for physical characteristics such as height and weight (~ 0.20) (Plomin & Deary, 2014). In twin studies such as ours and GHCA that do not also include parental data, nonadditive genetic variance could be masked by assortative mating, and there is some evidence that this is the case for intelligence (Vinkhuyzen, van der Sluis, Maes, & Posthuma, 2012). If assortative mating were similar for high intelligence and the entire sample, it would not affect the interpretation of our results, which are based solely on the twin design. However, if assortative mating were greater for high intelligence, this could mask greater nonadditive genetic variance for high intelligence (Vinkhuyzen et al., 2012). We are not aware of any studies that have investigated whether assortative mating differs as a function of level of intelligence.

### 9.1 Environmental and genetic discontinuity

The GHCA study found a trend supporting the environmental Discontinuity Hypothesis, in that shared family environmental influence was somewhat greater for high intelligence. In the GHCA study, shared environment was estimated at 28% in the high intelligence group using liability–threshold modelling and 21% in the entire sample, although the difference was not nearly significant (95% confidence intervals were 0.19–0.37 and 0.17–0.25, respectively). In the present study, the results were 36% for high intelligence and 22% for the entire sample, with the difference again non-significant. The shared environmental estimate for high intelligence from DF extremes analysis was similar in the present study (37%), although DF extremes analysis is less comparable to the other analyses. The confidence intervals overlap substantially for all of these comparisons.

For these reasons, we conclude that high intelligence is caused by the same environmental factors responsible for the normal distribution of intelligence. However, it should be mentioned that the Continuity Hypothesis is essentially a null hypothesis of no difference between high intelligence and the normal distribution. Caution is warranted because insufficient power biases results in favour of the Continuity Hypothesis. Nonetheless, the similarity of results from the GHCA study with 11,000 twin pairs and the present study with 9000 twin pairs affords strong, if not definitive, support for the Continuity Hypothesis.

As mentioned in Introduction, an important qualification for all of these conclusions is that more extreme cut-offs might yield different results. The GHCA study selected the top 15% of the distribution (although a case-control study with more extreme cut-offs is underway; Spain et al., in preparation), and the present study the top 5%. These cut-offs balance sample size and power. Twin studies are unlikely to reach adequate power using Galton's (1869) cut-offs of .025% for “eminent” and .0001% for “illustrious.” If 1% of births are twins, a population of 80 million would be needed to obtain a mere 200 pairs of twins above the .025% cut-off. However, molecular genetic studies could be useful even for extreme cut-offs, in part because they do not require special populations such as twins.

### 9.2 Positive genetics

Nothing would advance genetic research on intelligence more than identifying some of the genes responsible for its substantial heritability. We now know that many genes of very small effect are responsible for the heritability of intelligence, as is the case for all common disorders and complex dimensions in the life sciences (Plomin & Deary, 2014). Nonetheless a polygenic score that adds up the effects of many genes of small effect size would provide a strong test of the prediction from the Continuity Hypothesis that genes associated with normal variation in intelligence will also be associated with high intelligence. It could also be used to test the Continuity Hypothesis for very high cut-offs.

If the Continuity Hypothesis is correct, high intelligence represents the positive end of a normal distribution. In contrast, most genome-wide association research has focused on the negative effects of genes on disorders, diseases, and disabilities (Visscher, Brown, McCarthy, & Yang, 2012). For intelligence, the problematic end of the distribution has also become a focus of research, as rare non-inherited mutations are emerging as a major source of severe intellectual disability (Ellison et al., 2013). Genetic exploration of the positive tail of normally distributed traits such as high intelligence is important conceptually because it moves away from the notion that we are all the same genetically except for rogue mutations that cause disorders, diseases and disabilities. The term positive genetics has been used to highlight genetic research on the positive end of distributions (Plomin et al., 2009).

The normal phenotypic distribution of intelligence makes it an obvious target for investigating the positive as well as negative extremes. Another possibly important feature of intelligence is that, like athletic ability, it is assessed as maximal performance, in contrast to other behavioural domains such as psychopathology and personality that involve typical behaviour. However, the larger significance of positive genetics is that these phenotypic considerations about the positive pole of the normal distribution have far-reaching implications for genomics. Polygenic scores created from genome-wide association studies are normally distributed, for disorders as well as for dimensions. In other words, polygenic scores have a positive pole with just as many people as the negative pole, even though the spotlight is typically on the negative end of the distribution of genetic “risk.” This normal distribution of polygenic scores implies that at the level of DNA variation there are no common disorders, only normally distributed quantitative traits (Plomin et al., 2009).

Positive genetics and the Continuity Hypothesis have practical as well as conceptual implications for intelligence, for example, for identifying genes associated with intelligence. Rather than using the brute force strategy of getting ever-larger samples of unselected individuals to narrow the “missing heritability” gap (Plomin & Simpson, 2013), selecting individuals of high intelligence might increase power for gene-hunting based on the simple hypothesis that high-intelligence individuals are enriched for intelligence-enhancing alleles and harbour few intelligence-depleting alleles. In other words, intellectual development can be disrupted by any and many mutations, including non-inherited (*de novo*) mutations, but high intelligence requires that everything works correctly. This hypothesis provided the rationale for a genome-wide case-control association study for cases with extremely high intelligence (IQ > 150) compared to unselected control individuals (Spain et al., in preparation). However, in an initial report, this design does not appear to have found richer results either for identifying individual DNA variants, or for genomic approaches such as comparing the total number of rare variants (which generally have negative effects and might be expected to occur less frequently in the high-intelligence sample). Nonetheless, it is early days for the use of high-intelligence samples to increase power for gene-hunting.

Positive genetics raises the question: who are the people at the positive end of the polygenic distribution of “risk” for disorders? Are they merely individuals at low risk for problems, or do they have unusual positive traits? Thinking positively begins by thinking quantitatively — about “dimensions” rather than “disorders” and about genetic “variability” rather than genetic “risk.” Intelligence makes it easy to think positively.

## Figures and Tables

**Table 1:** Model-fitting results for whole twin sample. Results are additive genetic (A), shared environmental (C) and residual (E; i.e., non-shared environment and error) components of variance, with 95% confidence intervals.

Variance components (95% confidence intervals)			Sample (numbers of pairs)	
A	C	E	MZ	DZ
0.58 (0.53–0.63)	0.22 (0.17–0.27)	0.20 (0.19–0.21)	3039	3196

**Table 2:** Concordances. Concordance is shown both pairwise (the proportion of concordant pairs) and probandwise (the proportion of probands whose twin/sibling is also a proband).

	Number of pairs			Concordance	
	Total	Concordant	Discordant	Pairwise	Probandwise
MZ twins	185	54	131	0.29	0.45
DZ twins	196	28	168	0.14	0.25
Non-twin siblings	28,339	3302	25,037	0.12	0.21

**Table 3:** Tetrachoric correlations. *N* = 6235 twin pairs.

	Tetrachoric correlation (95% confidence interval)	Std. error
MZ twins	0.78 (0.71–0.84)	0.05
DZ twins	0.56 (0.45–0.66)	0.08

**Table 4:** Liability–threshold model-fitting results. Results are additive genetic (A), shared environmental (C) and residual (E; i.e., non-shared environment and error) components of variance. *N* = 6235 twin pairs.

Variance components (95% confidence intervals)		
A	C	E
0.42 (0.17–0.68)	0.36 (0.12–0.57)	0.22 (0.16–0.30)

**Table 5:** DF extremes model-fitting results. Results are additive genetic (A), shared environmental (C) and residual (E; i.e., non-shared environment and error) components of variance. *N* = 6235 twin pairs.

Group ACE components (95% confidence intervals)		
A	C	E
0.40 (0.28–0.52)	0.37 (0.27–0.46)	0.23 (0.19–0.27)
